# Calcium Chloride Treatment Enhances Antigen Production in Foot-and-Mouth Disease Vaccines for Serotypes SAT1 and SAT3

**DOI:** 10.3390/vaccines12030231

**Published:** 2024-02-23

**Authors:** Dohyun Kim, Sun Young Park, Gyeongmin Lee, Eun-Sol Kim, Jong-Sook Jin, Jae Young Kim, SooAh Lee, Jong-Hyeon Park, Young-Joon Ko

**Affiliations:** Center for Foot-and-Mouth Disease Vaccine Research, Animal and Plant Quarantine Agency, 177 Hyeoksin 8-ro, Gimcheon 39660, Republic of Korea; doh936@korea.kr (D.K.); sun3730@korea.kr (S.Y.P.); lgm6004@korea.kr (G.L.); kesol13@korea.kr (E.-S.K.); in75724@korea.kr (J.-S.J.); ivorikim@korea.kr (J.Y.K.); 0902lsa@korea.kr (S.L.); parkjhvet@korea.kr (J.-H.P.)

**Keywords:** foot-and-mouth disease virus, SAT, antigen, calcium, medium

## Abstract

Foot-and-mouth disease (FMD) is a highly contagious viral infection causing acute and severe vesicular lesions in cattle and pigs, which has prompted global vaccination policies. This study presents a technique for enhancing antigen yield in SAT1 BOT and SAT3 ZIM by treatment with calcium chloride (CaCl_2_). We tested changes in cell viability in BHK-21 suspension cells treated with varying concentrations of CaCl_2_. The optimal CaCl_2_ concentration was determined based on antigen yield. The timing of CaCl_2_ supplementation relative to FMD virus inoculation was tested. Finally, the optimal medium for antigen production was identified. We observed a concentration-dependent decrease in BHK-21 cell viability at >7.5 mM CaCl_2_. A CaCl_2_ concentration of 3 mM yielded the most antigens. CaCl_2_ supplementation relative to FMD virus infection was optimal 2 h before or with viral inoculation. CD-BHK 21 medium supplemented with CaCl_2_ was the most productive medium. Specifically, SAT1 BOT and SAT3 ZIM showed improved antigen production in CD-BHK 21 medium with 3 mM CaCl_2_, while Provero-1 and Cellvento BHK-200 media showed no significant enhancement. Overall, CaCl_2_ supplementation enhanced FMD antigen productivity. This study provides a useful framework for enhancing antigen production efficiently in the FMD vaccine industry.

## 1. Introduction

Foot-and-mouth disease (FMD) is an acute contagious disease affecting cows, pigs, and goats that causes vesicles on the mouth, teats, and hooves [1,2]. The etiologic agent is the FMD virus (FMDV, genus: *Aphthovirus*, family: *Picornaviridae*), which is divided into seven serotypes: A, O, C, Asia1, and South African Territories 1, 2, and 3 (SAT1, SAT2, and SAT3), and there is no known cross-immunity between serotypes [3]. Because FMDV is highly contagious, outbreaks are usually controlled by vaccination. In South Korea, a large-scale outbreak of FMD occurred in 2010–2011, causing massive economic damage, and since then, the government has implemented vaccination policies nationwide [4].

Effective FMD vaccines should elicit potent immune and antigenic responses similar to those with locally circulating FMDV [5]. Several affected countries, including South Korea, are developing region-specific FMD vaccine strains to address local contexts [6,7,8,9,10,11]. FMD vaccine strain research has explored virus strains in cattle, buffalo, goats, and pigs capable of mass production in baby hamster kidney (BHK-21) cells [12]. However, this process is time-consuming, costly, and antigen production may fail even after obtaining FMDVs adapted to BHK-21 cells. These limitations retard FMD vaccine research and development, especially because high antigen concentrations are needed to produce effective FMD vaccines [13]. Given the global impact of FMD, a cost-effective FMD antigen production technique is needed to support vaccine manufacturing and policy frameworks.

FMDV is composed of structural proteins that make up the capsid and single-stranded RNA inside the capsid. When the FMDV enters a cell and viral replication occurs, the structural proteins VP0, VP3, and VP1 form protomers and pentamers, and then 12 pentamers assemble to form the icosahedral FMDV. Finally, during RNA entry into the capsid, VP0 is cleaved into VP4 and VP2, resulting in the intact form of FMDV [14]. The main component of the FMD vaccine is the virus particle (146S), which is degraded to the pentamer 12S by external factors such as heat or pH, resulting in a more than 100-fold decrease in immunogenicity [15]. Therefore, it is very essential to mass-produce FMDV particles in an intact form (146S) during the production of FMD vaccine antigen. In addition, since the quantification of FMD vaccine antigen requires the measurement of virus particles rather than subunit proteins, the 146S fraction should be quantified using an ultracentrifuge or size-exclusion high-performance liquid chromatography (SE-HPLC).

Other research regarding calcium reported that plasmid-loaded calcium phosphate nanoparticles can activate dendritic cells better than free biomolecules in solution and can be applied to transfection with relevant plasmid DNA utilizing high affinity to nucleic acids [16]. In addition, polycations such as polybrene, protamine, DEAE-dextran and poly-L-lysine play roles by interrupting the repulsion between negative charges between adenoviruses and the cell surfaces [17].

In addition, it has been reported that divalent transition metal ions contribute to stabilization by forming salt bridges at the interpentameric interface of the FMDV capsid, and the effect is known to be dependent on FMDV strains [18]. We evaluated the stability of domestic FMDVs by treating them with divalent metal ions and found that calcium ions only improved the stability of both serotype O and serotype A viruses. The stabilizing effect of calcium ions on vaccine antigens prompted us to initiate this study.

The current study investigated the effect of calcium chloride (CaCl_2_) supplementation on FMD antigen production. We also determined the optimal reaction conditions and CaCl_2_ treatment parameters to maximize FMD antigen productivity. To the best of our knowledge, this is the first report of FMD vaccine antigen productivity being enhanced through supplementation with CaCl_2_.

## 2. Materials and Methods

### 2.1. Cells, Viruses, Calcium Chloride, and Titration

BHK-21 adherent cells (ATCC) were cultured in Dulbecco’s modified Eagle’s medium (Thermo Fisher Scientific, Waltham, MA, USA) supplemented with 10% fetal bovine serum (pH 7.4) in an incubator at 37 °C with 5% CO_2_. BHK-21 suspension cells were cultured in CD-BHK 21 (Lonza, Basel, Switzerland), Provero-1 serum-free (Lonza), or Cellvento BHK-200 production medium (Merck, Darmstadt, Germany) in a shaking incubator at 37 °C, 110 rpm, and 5% CO_2_. The BHK-21 suspension cells were established by the Animal and Plant Quarantine Agency (APQA) and the Korea Research Institute of Bioscience and Biotechnology in the Republic of Korea. Cell numbers and viability were analyzed via the trypan blue exclusion method using an automated cell counter (Vi-Cell XR; Beckman Coulter, Brea, CA, USA). Calcium chloride was purchased from Sigma-Aldrich (St. Louis, MO, USA), dissolved in distilled water, and used after preparing a 300 mM stock. FMDV SAT1 BOT and SAT3 ZIM were isolated from the infectious clones, as previously described [9]. FMDV infection and culture were performed at APQA’s biosafety level 3 facility. The titer of FMDV was measured in adherent BHK-21 cells. The viral titers were calculated using the Reed and Muench method at a 50% tissue culture infective dose (TCID_50_) [19].

### 2.2. Quantification of FMD Vaccine Antigen

The harvested FMDV supernatant was vortexed with chloroform [1:1.5 (*v*/*v*), Merck] for 5 min, centrifuged at 4000× *g* rpm for 15 min at 4 °C, and the supernatant was collected and treated with benzonase (Sigma-Aldrich) at a final concentration of 0.025 units/µL and reacted at 37 °C for 1 h. The supernatant was collected by centrifugation at 16,000× *g* for 10 min at 4 °C and loaded for size-exclusion high performance liquid chromatography (SE-HPLC)-based quantification of the FMD vaccine antigen as previously described [20,21]. Briefly, the analysis was carried out on a TSKgel G4000PWXL (300 mm × 7.8 mm I.D.) column (TOSOH Bioscience, Tokyo, Japan) fitted with a TSKgel PWXL Guardcol (40 mm × 6.0 mm) guard column (TOSOH Bioscience, Tokyo, Japan) using an Agilent 1260 Infinity II system (Agilent Technologies, Santa Clara, CA, USA), with a variable wavelength detector operating at 254 nm. The mobile phase was 30 mM Tris-HCl and 400 mM NaCl (pH 8.0), and the flow rate was 0.5 mL/min. The area under the target peak was calculated to quantify 146S antigens according to a previous study [22].

### 2.3. Cell Viability Assay of BHK-21 Suspension Cells Treated with CaCl_2_

BHK-21 suspension cells (3 × 10^5^ cells/mL) were cultured in CD-BHK 21 medium with dissolved CaCl_2_ at 60, 30, 15, 7.5, 3.75, 1.8, and 0.9 mM for 3.5 d. The culture was clarified by centrifugation at 2000× *g* for 3 min at 3 °C. The cells were resuspended in CD-BHK 21 medium supplemented with CaCl_2_ and further cultured for 24 h at 37 °C before cell viability was evaluated using the Vi-CELL XR system (Beckman Coulter).

### 2.4. Determining Optimal CaCl_2_ Concentration for FMD Vaccine Antigen Production

BHK-21 suspension cells were seeded in 30 mL of CD-BHK 21 medium at 3 × 10^5^ cells/mL and cultured at 37 °C for 3.5 d. The cell culture supernatant was removed by centrifugation at 2000× *g* for 3 min at 4 °C and then replaced with fresh CD-BHK 21 medium. Cells were infected with FMDV SAT1 BOT or SAT3 ZIM at a multiplicity of infection (MOI) of 0.01 and CaCl_2_ was concurrently added to achieve final concentrations of 1, 3, 10, or 30 mM. The cells were cultured in a shaking incubator at 37 °C with 5% CO_2_ for 24 h. The culture was subjected to two freeze–thaw cycles and centrifuged at 4000× *g* rpm for 10 min at 4 °C to collect the supernatant, from which the virus titer and 146S antigen were measured using titration and SE-HPLC, respectively.

### 2.5. Determining Optimal Timing for CaCl_2_ Addition in FMD Vaccine Antigen Production

BHK-21 suspension cells were seeded in 30 mL CD-BHK 21 medium at 3 × 10^5^ cells/mL and cultured at 37 °C for 3.5 d. The cell culture supernatant was removed by centrifugation at 2000× *g* and 4 °C for 3 min, and then replaced with fresh CD-BHK 21 medium. The cells were infected with FMDV SAT1 BOT or SAT3 ZIM at 0.01 MOI. CaCl_2_ was added at 2 h before, simultaneously with, 2 h post infection (hpi), or 24 hpi. The cells were cultured in a shaking incubator at 37 °C and 5% CO_2_ for 24 h. The culture was subjected to two freeze–thaw cycles, centrifuged at 4000× *g* rpm and 4 °C for 20 min to collect the supernatant, from which the virus titer and 146S antigen were measured using titration and SE-HPLC, respectively.

### 2.6. Determining Optimal Medium for CaCl_2_-Modified FMD Vaccine Antigen Production

BHK-21 suspension cells (3 × 10^5^ cells/mL) were seeded in 30 mL of CD-BHK 21, Provero-1, or Cellvento medium and cultured at 37 °C for 3.5 d. Cells were infected with SAT1 BOT or SAT3 ZIM viruses at 0.01 MOI and incubated at 37 °C for 24 h. CaCl_2_ was added at the time of virus inoculation to achieve a final concentration of 3 mM. Cells were harvested 24 hpi, the supernatant was collected by centrifugation at 4000× *g* rpm and 4 °C for 20 min, and virial titers and 146S antigen were quantified using titration and SE-HPLC, respectively.

### 2.7. Statistical Analysis

Differences between groups were assessed using unpaired *t*-tests performed in GraphPad Prism Software (version 5.0, GraphPad Software, La Jolla, CA, USA). A *p*-value < 0.05 was considered significant.

## 3. Results

### 3.1. Cell Viability Assay of BHK-21 Suspension Cells Treated with CaCl_2_

**Figure 1:**
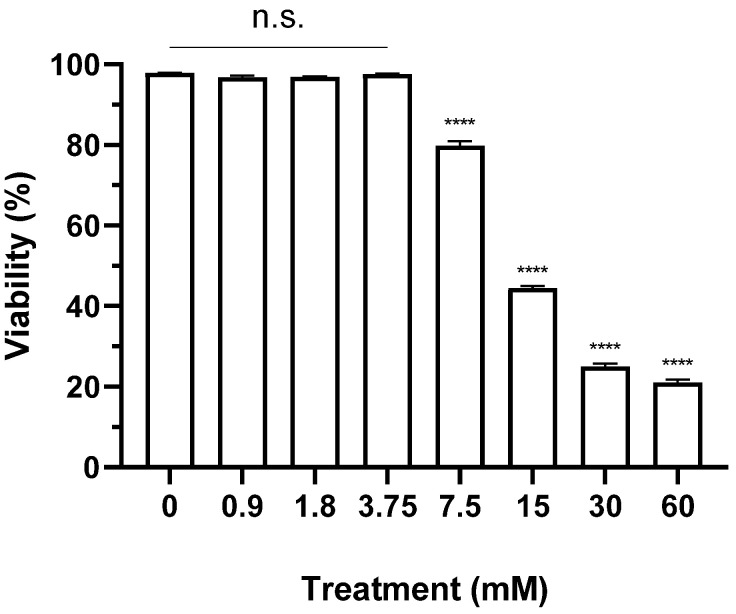
Cytotoxicity of CaCl_2_ in BHK-21 suspension cell line for FMD vaccine antigen production. BHK-21 cells were treated with 60, 30, 15, 7.5, 3.75, 1.8, and 0.7 mM calcium chloride and cultured for 24 h. Cell viability was observed using Vi-CELL XR Cell viability analyzer system. Each experiment was conducted three times, and the error bars indicate standard deviations (SDs) from the mean. n.s., non-significant; **** *p* < 0.001; unpaired *t*-test.

The viability of BHK-21 suspension cells cultured in medium with <3.75 mM CaCl_2_ was 96–98%, showing no significant difference compared to the untreated group (Figure 1). BHK-21 suspension cell viability began to significantly decrease at ≥7.5 mM compared to that of the untreated group. Cells treated with 60 mM CaCl_2_ showed a viability of 20%.

### 3.2. Optimal CaCl_2_ Concentration for FMD Vaccine Antigen Production

**Figure 2:**
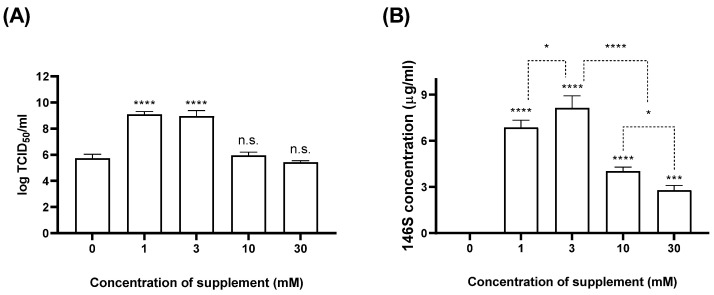
Comparison of SAT1 BOT virial titer and antigen productivity at various CaCl_2_ concentrations. BHK-21 suspension cells were treated with 30, 10, 3, and 1 mM CaCl_2_ simultaneously (MOI: 0.01) and cultured for 24 h at 37 °C. The virial titer (**A**) and antigen productivity (**B**) were measured by virus titration and size-exclusion high-performance liquid chromatography (SE-HPLC). Each experiment was conducted three times, and the error bars indicate SDs from the mean. n.s., non-significant; * *p* < 0.05, *** *p* < 0.005, **** *p* < 0.001; unpaired *t*-test.

**Figure 3:**
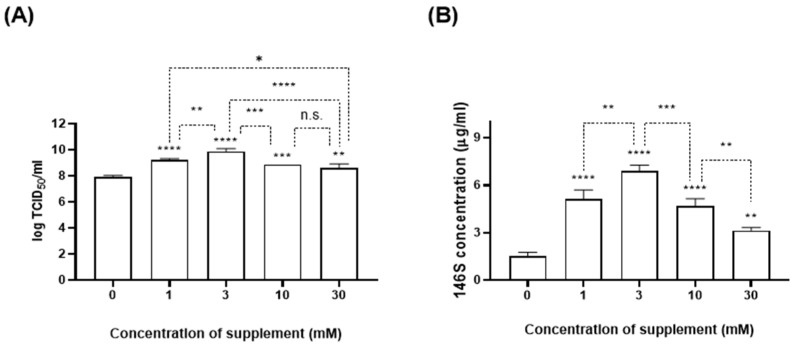
Comparison of SAT3 ZIM virial titer and antigen productivity at various CaCl_2_ concentrations. BHK-21 suspension cells were treated with 30, 10, 3, and 1 mM CaCl_2_ simultaneously (MOI: 0.01) and cultured for 24 h at 37 °C. The virial titer (**A**) and antigen productivity (**B**) were measured by virus titration and SE-HPLC. Each experiment was conducted three times, and the error bars indicate SDs from the mean. n.s., non-significant; * *p* < 0.05, ** *p* < 0.01, *** *p* < 0.005, **** *p* < 0.001; unpaired *t*-test.

BHK-21 suspension cells were infected with SAT1 BOT and SAT3 ZIM (MOI: 0.01) and concurrently treated with various concentrations of CaCl_2_. The culture supernatant was collected at 24 hpi, and the titer and antigen quantity were measured (Figure 2 and Figure 3). When 1 or 3 mM CaCl_2_ was added during the SAT1 BOT infection, the virus titer reached 9 log TCID_50_/mL. However, 10 mM or 30 mM additions achieved titers comparable to those in the negative control group (5.5 log TCID_50_/mL) (Figure 2A). While no antigen was detected in the negative control group, the CaCl_2_-treated groups exhibited antigen yields of ≥2.7 µg/mL (Figure 2B). The group treated with 3 mM CaCl_2_ showed the highest antigen yield (up to 8.13 µg/mL). With 1 or 3 mM CaCl_2_ during SAT3 ZIM infection, a virus yield of 10 log TCID_50_/mL was obtained (a 100-fold increase compared to the control group). The viral titers in groups treated with 10 or 30 mM CaCl_2_ increased approximately 10-fold compared to those of the negative control group (Figure 3A). All CaCl_2_-treated groups exhibited an antigen productivity of ≥3.1 µg/mL. The 3 mM CaCl_2_-treated group recorded the highest antigen yield (6.89 µg/mL), demonstrating superior antigen production efficiency (Figure 3B). The results indicate an approximately 4.6-fold improvement in antigen yield compared to the negative control group.

### 3.3. Optimal Timing for CaCl_2_ Addition in FMD Vaccine Antigen Production

**Figure 4:**
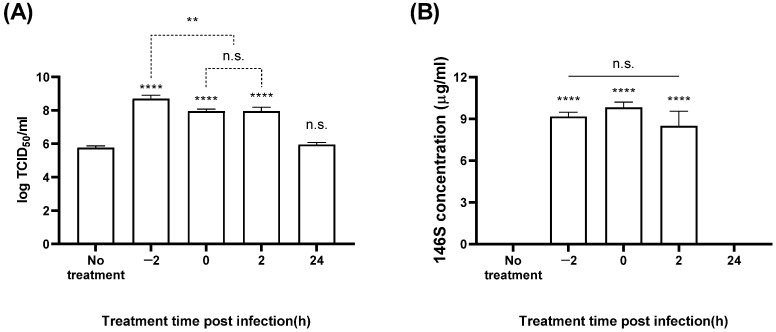
Antigen production in SAT1 BOT according to the timing of CaCl_2_ addition. The BHK-21 suspension cells were treated with 3 mM calcium chloride at 2 h before FMDV infection (MOI: 0.01), at FMDV infection, 2 hpi, and 24 hpi. The virial titer (**A**) and antigen productivity (**B**) were determined in the culture supernatant after 24 h. Each experiment was conducted three times, and the error bars indicate SDs from the mean. n.s., not significant; ** *p* < 0.01, **** *p* < 0.001; unpaired *t*-test.

**Figure 5:**
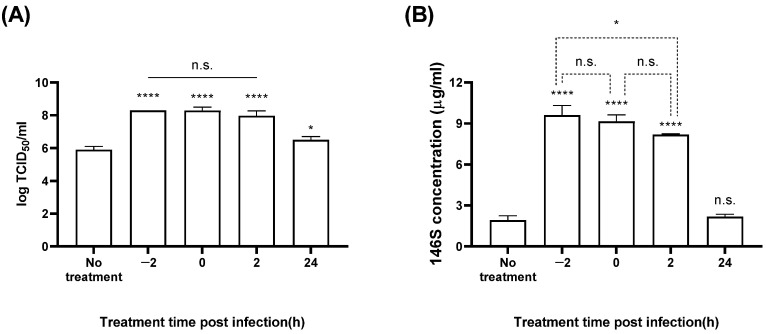
Antigen production in SAT3 ZIM according to the timing of CaCl_2_ addition. The BHK-21 suspension cells were treated with 3 mM calcium chloride at 2 h before FMDV infection (MOI: 0.01), at FMDV infection, 2 hpi, and 24 hpi. The virial titer (**A**) and antigen productivity (**B**) were determined in the culture supernatant after 24 h. Each experiment was conducted three times, and the error bars indicate SDs from the mean. n.s., non-significant; * *p* < 0.05, **** *p* < 0.001; unpaired *t*-test.

BHK-21 cells were treated with 3 mM CaCl_2_ 2 h before FMDV infection (−2 hpi), at the time of infection (0 hpi), 2 hpi, or 24 hpi; the titer and antigen quantity of the culture medium were measured (Figure 4 and Figure 5). With SAT1 BOT, CaCl_2_ treatment at −2 hpi showed the best titers (8.7 log TCID_50_/mL) (Figure 4A), representing an approximately 1000-fold increase compared to the control group. No antigen was produced with SAT1 BOT without CaCl_2_ treatment; cells treated with CaCl_2_ at 0 hpi exhibited the highest antigen yield (9.8 µg/mL) (Figure 4B). The groups treated with CaCl_2_ at −2 or 2 hpi showed antigen yields of 9.2 and 8.5 µg/mL, respectively, with no significant difference compared to the 0 hpi group.

With SAT3 ZIM, the groups treated with CaCl_2_ at −2, 0, or 2 hpi all showed titers of ~8.2 log TCID_50_/mL (Figure 5A). Compared to the untreated group, CaCl_2_ treatment corresponded with an approximately 250-fold increase in titers. The antigen yield in the negative control group was 1.9 µg/mL, whereas CaCl_2_ treatments at −2, 0, or 2 hpi showed antigen yields of 9.6, 9.2, and 8.2 µg/mL, respectively (Figure 5B). There was no significant difference between the –2 and 0 hpi groups or the 0 and 2 hpi groups.

### 3.4. Optimal Medium for CaCl_2_ Addition in FMD Vaccine Antigen Production

**Figure 6:**
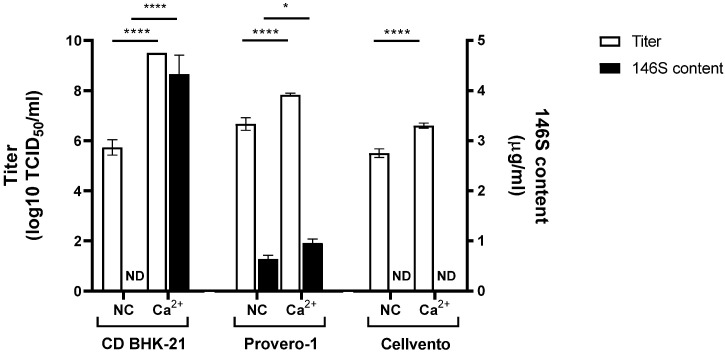
Effect of CaCl_2_ addition on SAT1 BOT virus titer and antigen production in various media. Three mM of CaCl_2_ was added at 0 hpi with SAT1 BOT in 30 mL of CD-BHK 21, Provero-1, or Cellvento medium. White bars indicate viral titer. Black bars indicate FMD vaccine antigen content measured using SE-HPLC. Each experiment was conducted three times, and the error bars indicate SDs from the mean. ND, not detected, * *p* < 0.05, **** *p* < 0.001; unpaired *t*-test.

**Figure 7:**
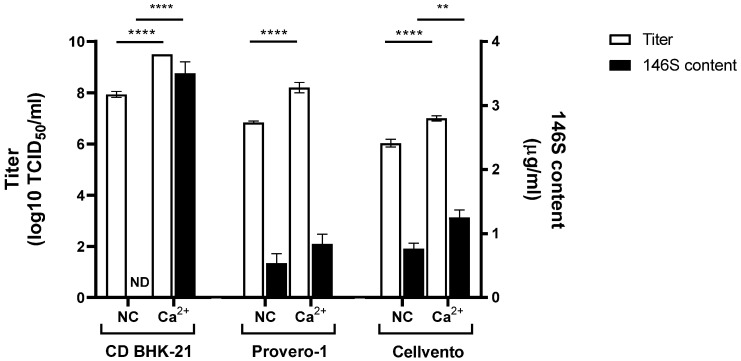
Effect of CaCl_2_ addition on SAT3 ZIM virus titer and antigen production in various media. Three mM of CaCl_2_ was added at 0 hpi with SAT3 ZIM in 30 mL of CD-BHK 21, Provero-1, or Cellvento medium. White bars indicate viral titer. Black bars indicate FMD vaccine antigen content measured using SE-HPLC. Each experiment was conducted three times, and the error bars indicate SDs from the mean. ND, not detected, ** *p* < 0.01, **** *p* < 0.001; unpaired *t*-test.

We examined the optimal medium for producing FMD vaccine antigens with CaCl_2_ stimulation with both SAT1 BOT and SAT3 ZIM (Figure 6 and Figure 7). With SAT1 BOT, CD-BHK 21 medium produced a titer of 5.7 log TCID_50_/mL, with no detectable antigen production. Conversely, CD-BHK 21 supplemented with CaCl_2_ produced a virus with a titer of 9.5 log TCID_50_/mL, accompanied by an antigen yield of 4.3 µg/mL (Figure 6). In the Provero-1 and Cellvento media, CaCl_2_ supplementation significantly enhanced the virus titers compared to the control group; however, antigen production was either below 1 µg/mL or not evident.

With SAT3 ZIM, CD-BHK 21 produced a virus titer of 7.9 log TCID_50_/mL, with no antigens produced. In contrast, CaCl_2_-supplemented CD-BHK 21 medium produced 9.5 log TCID_50_/mL, and an antigen yield of 3.5 µg/mL. Notably, both the titer and antigen productivity showed a significant increase compared to those in the CaCl_2_ untreated group (Figure 7). However, antigen yield (below 0.84 µg/mL) with SAT3 ZIM did not differ when using either Provero-1 or CaCl_2_-supplemented Provero-1 medium. The virus titer with SAT3 ZIM in CaCl_2_-supplemented Cellvento medium was 7 log TCID_50_/mL, with an antigen yield of 1.25 µg/mL. Notably, this represented a tenfold increase in virus titer compared to that with Cellvento alone and a 1.6-fold increase in antigen production yield.

The value of raw data for virus titier and 146S content used in all graph are presented in the Supplementary Materials.

## 4. Discussion

Efficient production of FMD vaccine antigens is crucial for supporting FMD vaccination policies as well as research and development. This study proposes a method for enhancing the yield of FMD vaccine antigens per unit volume by adding CaCl_2_ during the antigen production process in SAT1 BOT and SAT3 ZIM FMD vaccine strains.

Firstly, we determined that the maximum non-toxic concentration of CaCl_2_ for BHK-21 suspension cells for antigen production was 3.75 mM (Figure 1). The virial titer and antigen productivity of SAT1 BOT and SAT3 ZIM FMD vaccine strains were enhanced with the addition of CaCl_2_, especially at 3 mM (Figure 2 and Figure 3). The increase in calcium ions 2–4 h after polio virus infection, which induces the expression of the non-structural protein 2BC of the polio virus, indicates viral replication [23,24]. However, the present study is the first to report that the addition of calcium ions enhanced virus titer and antigen productivity during FMD vaccine antigen production.

We found that adding CaCl_2_ at −2, 0, and 2 hpi significantly improved the virus titer and vaccine antigen productivity in SAT1 BOT and SAT3 ZIM FMDVs (Figure 4 and Figure 5). These results indicate that calcium ions affect cellular metabolism, cell–virus interactions, and intracellular virus replication. Noticeably, unlike the titers, we observed distinct variations in antigen productivity depending on the timing of CaCl_2_ addition.

Finally, we confirmed that CaCl_2_ supplementation increased FMD vaccine antigen production in various media (Figure 6 and Figure 7). CaCl_2_ addition only had a noticeable effect on antigen production in SAT1 BOT and SAT3 ZIM when using CD-BHK 21 medium. In previous reports, variations in titers and antigen productivity were observed depending on the type of production medium used in FMD vaccine antigen production [25,26]. The mechanism behind the enhanced productivity of FMD antigens in CD-BHK 21 medium, possibly due to interactions between its components and CaCl_2_, remains unknown. Our findings emphasize the critical importance of medium selection in improving FMD vaccine antigen production with CaCl_2_ supplementation.

Our findings go a long way toward elucidating how CaCl_2_ impacts the titers and antigen productivity of the FMDV. Calcium ions enhance the binding between the serine in integrin αvβ6 receptors present on the host cell and the aspartate in RGD motifs of the FMDV [27,28]. In hepatitis A (of the picornaviruses), 2 mM calcium ions strengthened cell–virus binding [29]. We observed an enhancement in FMDV titers at a similar concentration of calcium ions (Figure 2A and Figure 3A). The FMDV invades the cell and translates the 2B non-structural protein, expressed in the Golgi complex, endoplasmic reticulum (ER), and cytosol [30]. The 2B protein releases calcium ions within the Golgi complex and ER, disrupting the protein trafficking pathway and prompting the host cell to uptake external calcium ions through the calcium ion channel (Orai1) or viroporin [31,32,33]. The increased intracellular free calcium ion level boosts mitochondrial ATP production [31]. Consistently, our experiments showed that the addition of calcium ions during the entry and replication stages of the FMDV promoted viral potency (Figure 4 and Figure 5). However, vaccine antigen productivity does not correspond to virus titers. The untreated SAT1 BOT- and SAT3 ZIM groups did not exhibit low titers for antigen production (Figure 2, Figure 3, Figure 4, Figure 5 and Figure 6). SAT1 BOT and SAT3 ZIM FMDVs were previously reported with antigen yields of 7 log TCID_50_/mL [10,11]. The 146S fraction must be quantified using ultracentrifugation or HPLC because the quantification of FMD vaccine antigen requires the measurement of virus particles rather than subunit proteins. Therefore, even if viral titers are detected, the antigen may not be detected. This is because viral titers are measured by infecting cells with the virus and amplifying it, whereas the amount of 146S is measured by ultracentrifugation, or HPLC, to determine the amount of virus particles present. In the case of FMDV type Asia1 [34], the viral titers are equivalent, but the amount of antigen corresponding to the viral particles (146S) varies greatly depending on various conditions, such as FMDV concentration and viral infection time. Meanwhile, the efficacy of the FMD vaccine depends on the 146S intact virus particle content rather than the virus titer. Other groups reported similar results that high viral titers but low antigen amounts lead to low immunogenicity in animals [35].

SAT1 BOT and SAT3 ZIM, which were used in this study, have been previously shown to provide viral defense in pigs, in addition to immunotypic performance in pigs and cattle [9]. In addition to the results showing that calcium treatment improved the stability of the physically unstable type O FMDV, we also have data showing that the immunogenicity in pigs of the calcium-treated and untreated experimental groups for type O FMDV was the same, indicating that the calcium treatment did not exert any negative effect on the vaccine-induced immunogenicity in pigs. Extrapolating from this, it is unlikely that there is a difference in immunogenicity between SAT1 and SAT3 viruses with or without calcium addition. We hypothesized that, upon completion of replication, calcium ions enhance the stability of the FMDV capsid, thereby increasing antigen productivity. Subsequent studies should verify the immunogenicity of antigens produced through the calcium ion addition technique in experimental animals. Enhancing antigen productivity requires not only that the system promote FMDV replication but also antigen stability. Fortunately, calcium ions bind to Glu6 in the VP2 structural protein located on the icosahedral 3-fold axes of the FMDV, forming an ion bridge and contributing to the enhancement of thermal stability [36,37,38]. The precise mechanism by which calcium ions influence the formation of FMD vaccine antigens requires further investigation.

## 5. Conclusions

The addition of CaCl_2_ enhanced FMD vaccine antigen productivity not only in FMDVs that were previously unproductive but also showed improved viral titers. To my knowledge, this is the first report that a simple treatment with calcium chloride yields a dramatic increase in the amount of FMD vaccine antigen, and this will be a breakthrough with immediate implications for the field of FMD vaccine production. Our study can inform the development of more efficient strategies for producing FMD vaccines that can rapidly meet changing demands with continued disease outbreaks.

## Data Availability

The original contributions presented in the study are included in the article/Supplementary Material, further inquiries can be directed to the corresponding author/s.

## Supplementary Materials

The following supporting information can be downloaded at: https://www.mdpi.com/article/10.3390/vaccines12030231/s1, Table S1: Comparison of SAT1 BOT virial titer and antigen productivity at various CaCl_2_ concentration, Table S2: Comparison of SAT3 ZIM virial titer and antigen productivity at various CaCl_2_ concentration, Table S3: Production efficiency of SAT1 BOT antigen according to the timing of CaCl_2_ addition, Table S4: Production efficiency of SAT3 ZIM antigen according to the timing of CaCl_2_ addition, Table S5: Effect of CaCl_2_ addition on SAT1 BOT virus titer and antigen production in CD-BHK, Provero-1, and Cellvento medium. Table S6: Effect of CaCl_2_ addition on SAT3 ZIM virus titer and antigen production in CD-BHK, Provero-1, and Cellvento medium.
