# Supplementary material for: Calcium Chloride Treatment Enhances Antigen Production in Foot-and-Mouth Disease Vaccines for Serotypes SAT1 and SAT3
Source: Vaccines (Basel). 2024 Feb 23;12(3):231. doi: 10.3390/vaccines12030231 (PMC10976096; doi:10.3390/vaccines12030231)
Supplement: Supplementary file 1 [file vaccines-12-00231-s001.zip › vaccines-2860246-supplementary.pdf]

Table S1. Comparison of SAT1 BOT virial titer and antigen productivity at various $CaCl_2$ concentration. BHK-21 suspension cells were treated with 30, 10, 3, 1 mM $CaCl_2$ simultaneously with 0.01 MOI of SAT1 BOT infection and cultured for 24 h at 37℃. The virial titer and antigen productivity were measured by virus titration and SE-HPLC.

Concentration of supplement (mM)	Viral titer (log $TCID_{50}$/mL)	146S concentration (μg/mL)
0	5.73 ± 0.31	0
1	9.10 ± 0.20	6.87 ± 0.47
3	8.97 ± 0.42	8.13 ± 0.80
10	5.97 ± 0.23	4.03 ± 0.26
30	5.43 ± 0.12	2.79 ± 0.31

Table S2. Comparison of SAT3 ZIM virial titer and antigen productivity at various $CaCl_2$ concentration. BHK-21 suspension cells were treated with 30, 10, 3, 1 mM $CaCl_2$ simultaneously with 0.01 MOI of SAT3 ZIM infection and cultured for 24 h at 37℃. The virial titer and antigen productivity were measured by virus titration and SE-HPLC.

Concentration of supplement (mM)	Viral titer (log $TCID_{50}$/mL)	146S concentration (μg/mL)
0	7.93 ± 0.12	1.50 ± 0.26
1	9.23 ± 0.12	5.14 ± 0.56
3	9.90 ± 0.20	6.89 ± 0.38
10	8.90 ± 0	4.69 ± 0.47
30	8.63 ± 0.31	3.15 ± 0.18

Table S3. Production efficiency of SAT1 BOT antigen according to the timing of $CaCl_2$ addition. The BHK-21 suspension cells were treated with 3 mM calcium chloride at the time of 2 h before FMDV infection, FMDV infection, 2 h post-infection, and 24 h post-infection. The cells were infected with SAT1 BOT FMDV at 0.01 MOI and cultured for 24 h. The culture supernatant was collected 24 h post-virus infection, and the virus titer and antigen productivity were determined by virus titration and SE-HPLC.

Treatment time post infection (h)	Viral titer (log $TCID_{50}$/mL)	146S concentration (μg/mL)
No treatment	5.77 ± 0.12	0
-2	8.70 ± 0.20	9.17 ± 0.31
0	7.97 ± 0.12	9.84 ± 0.38
2	7.97 ± 0.23	8.50 ± 1.05
24	5.97 ± 0.12	0

Table S4. Production efficiency of SAT3 ZIM antigen according to the timing of $CaCl_2$ addition. The BHK-21 suspension cells were treated with 3 mM calcium chloride at the time of 2 h before FMDV infection, FMD virus infection, 2 h post-infection, and 24 h post-infection. The cells were infected with SAT3 ZIM FMDV at 0.01 MOI and cultured for 24 h. The culture supernatant was collected 24 h post-virus infection, and the virus titer and antigen productivity were determined by virus titration and SE-HPLC.

Treatment time post infection (h)	Viral titer (log $TCID_{50}$/mL)	146S concentration (μg/mL)
No treatment	5.90 ± 0.20	1.91 ± 0.34
-2	8.30 ± 0.00	9.61 ± 0.70
0	8.30 ± 0.20	9.15 ± 0.48
2	7.97 ± 0.31	8.19 ± 0.06
24	6.50 ± 0.20	2.17 ± 0.18

Table S5. Effect of $CaCl_2$ addition on SAT1 BOT virus titer and antigen production in CD-BHK, Provero-1, and Cellvento medium. 3 mM calcium chloride was added at 0 h post-infection of SAT1 BOT in the 30 mL of CD-BHK, Provero-1, and Cellvento medium. The culture supernatant was collected 24 h post-virus infection, and the virus titer and antigen productivity were determined by virus titration and SE-HPLC.

Production media	Viral titer (log $TCID_{50}$/mL)		146S concentration (μg/mL)	
	NC	3 mM $CaCl_2$	NC	3 mM $CaCl_2$
CD-BHK	5.73 ± 0.31	9.50 ± 0.00	0	4.33 ± 0.38
Provero-1	6.67 ± 0.25	7.83 ± 0.06	0.64 ± 0.08	0.96 ± 0.08
Cellvento	5.50 ± 0.17	6.60 ± 0.10	0	0

Table S6. Effect of $CaCl_2$ addition on SAT3 ZIM virus titer and antigen production in CD-BHK, Provero-1, and Cellvento medium. 3 mM calcium chloride was added at 0 h post-infection of SAT3 ZIM in the 30 mL of CD-BHK, Provero-1, and Cellvento medium. The culture supernatant was collected 24 h post-virus infection, and the virus titer and antigen productivity were determined by virus titration and SE-HPLC.

Production media	Viral titer (log $TCID_{50}$/mL)		146S concentration (μg/mL)	
	NC	3 mM $CaCl_2$	NC	3 mM $CaCl_2$
CD-BHK	7.93 ± 0.12	9.50 ± 0.00	0	3.50 ± 0.18
Provero-1	6.83 ± 0.06	8.20 ± 0.20	0.54 ± 0.15	0.84 ± 0.15
Cellvento	6.03 ± 0.15	7.00 ± 0.10	0.76 ± 0.09	1.25 ± 0.12
